# Physiologic changes in microgravity may lead to unpredictable effects of spinal anesthesia

**DOI:** 10.3389/fphys.2026.1773665

**Published:** 2026-02-18

**Authors:** Siobhan Wagner, Matthew Turnock

**Affiliations:** 1 Department of Anesthesiology and Pain Medicine, University of Toronto, Toronto, ON, Canada; 2 Department of Anesthesiology, Pharmacology, and Therapeutics, University of British Columbia, Vancouver, ON, Canada

**Keywords:** baricity, cerebrospinal fluid, hemodynamics, intrathecal drug spread, microgravity, space medicine, spaceflight, spinal anesthesia

## Abstract

Regional techniques such as spinal anesthesia may offer advantages over general anesthesia for autonomous medical care during long-duration space missions, yet their behaviour in microgravity remains largely uncharacterised. On Earth, intrathecal anesthetic spread depends on baricity, posture, spinal curvature, and cerebrospinal fluid dynamics. Microgravity alters these determinants by diminishing gravity-dependent density gradients, modifying spinal geometry through paraspinal atrophy and elongation, and inducing cardiovascular and neurophysiologic adaptations that may affect block characteristics and hemodynamic tolerance. This Mini Review synthesises current evidence on spinal anesthetic mechanisms and spaceflight physiology to identify where terrestrial assumptions may fail in microgravity. Key knowledge gaps and research priorities are highlighted to inform the development of safe neuraxial anesthesia protocols for exploration-class missions.

## 1 Introduction

In recent years, growth in the global space exploration sector has taken off, with government agencies and private companies planning ventures ranging from commercial low-Earth orbit space stations to long-term habitation of Mars. As more people are exposed to microgravity for longer periods, there is a growing need to understand how severe sickness and injury will be managed during space flight. Because crew will operate at great distance from Earth with limited resupply and communication delays, the ability to perform surgery and anesthesia on board may be critical for survival. Traumatic injury has been identified by NASA as the highest medical concern for mission success, and EVAs, spacewalks and spacecraft repairs increase cumulative risk of trauma, musculoskeletal injury, hypoxia and decompression sickness (Billica et al., 1996; Kirkpatrick et al., 2009; Anderton et al., 2019). Extrapolating from the general population, at least one surgical emergency might be expected during a multi-year Mars mission with a crew of seven (Summers et al., 2005). Although this must be interpreted in the context of rigorous astronaut selection criteria and mission planning, it highlights that the capability to provide effective anesthesia becomes essential as more humans are exposed to microgravity and longer duration missions take place.

While surgery in microgravity has been explored in parabolic flight studies, research on anesthesia in space remains sparse, limited to a handful of studies on intravenous agents, regional techniques, cardiopulmonary resuscitation and airway management (Ball et al., 2010; Komorowski et al., 2013; Kiberd et al., 2024). In this Mini Review, a targeted, non-systematic literature search through January 2026 was conducted using PubMed/MEDLINE, Google Scholar, and the Cochrane Library, using the following search terms: microgravity, spaceflight, spinal and neuraxial anesthesia, intrathecal drug spread, cerebrospinal fluid, and space physiology. Given the absence of direct human data, evidence was synthesized narratively from terrestrial spinal anesthesia studies, spaceflight and analogue physiology research, and relevant reviews, with emphasis on mechanistic plausibility and relevance to microgravity rather than formal quality scoring.

### 1.1 Rationale for regional anesthesia in spaceflight

Thus far, anesthetic procedures have not been required during space flight and constrained resources in terms of mass, volume and power remain distinct challenges for future anesthetic use (Norfleet, 2000). Selecting an appropriate anesthetic technique in such an austere environment will depend on a variety of factors including the patient’s health status, the crew’s proficiency and the level of urgency. In microgravity, general anesthesia is comparatively less favorable because it necessitates airway instrumentation and controlled ventilation, while microgravity-induced cardiovascular deconditioning and altered pharmacodynamics may reduce physiologic tolerance to systemic anesthetic agents. A main consideration is the route of administration, as vapours may contaminate cabin atmosphere, unintentionally impacting other individuals and posing a potential fire hazard (Norfleet, 2000). Intravenous general anesthetics, such as ketamine, have been successfully administered in animal models during orbit (Campbell et al., 2005). However the volume and mass of medication required to provide a suitable duration of anesthesia may be prohibitive for long-duration missions for a low-occurrence event.

Regional and spinal anesthesia may be preferable to general anesthesia given that regional anesthesia allows the patient to remain conscious with less reliance on additional mechanical medical support. Around the world in resource-poor countries, regional and neuraxial anesthesia, including spinal anesthesia, have been administered by non-physicians, such as nurses and medical officers, many of whom acquire these skills on the job (Adesunkanmi, 1997; Campbell et al., 2004; Mellor, 2005; Schnittger, 2007; Jochberger et al., 2008; Newton and Bird, 2010; Martin et al., 2015). This utilisation highlights that proficiency in these techniques can be successfully imparted through training. In particular, as researchers predict that the main causes of surgical emergency during space exploration will be trauma, appendicitis, or cholecystitis, spinal anesthesia will be preferable as it is commonly used for procedures of the abdomen and below the umbilicus (Ball et al., 2012; Olawin and Das, 2024).

### 1.2 Determinants of intrathecal spread

Spinal anesthesia is delivered by an intrathecal injection of local anesthetic into the cerebrospinal fluid (CSF) surrounding the spinal cord, with the goal of achieving an adequate sensory and motor block while minimising the complications associated with excessive spread. Intrathecal distribution is influenced by a combination of procedural variables including injection speed, vertebral level, baricity, dose, and volume, as well as patient-related factors such as positioning. Other determinants, including spinal curvature and genetic or acquired anatomic variation, are less readily modifiable (Hocking and Wildsmith, 2004).

The relationship between the density of the CSF and local anesthetic, at body temperature, is also known as the medication’s baricity. Isobaric (“plain”) anesthetic solutions densities within the normal CSF range (approximately 1.0000–1.0006 g/mL) (Hocking and Wildsmith, 2004). In contrast, hyperbaric “heavy” anesthetic solutions, with greater density relative to CSF, distribute in a gravity-dependent manner, with patient position altering the gravity vector relative to the spinal cord and thereby influencing spread (Hocking and Wildsmith, 2004).

Removal of a sustained gravity vector fundamentally changes the factors governing intrathecal spread by eliminating the density-dependent layering that normally shapes anesthetic distribution on Earth. In the absence of this hydrostatic influence, mechanisms such as CSF pulsatility, cardiorespiratory pressure oscillations, and injection-related mixing are likely to assume greater relative importance. Under these conditions, intrathecal distribution is expected to reflect a larger contribution from diffusion and low-level convective transport rather than gravity-driven sedimentation, resulting in spread that may be more symmetric and less position dependent, but also more variable in extent and onset.

#### 1.2.1 Anatomic factors

**FIGURE 1:**
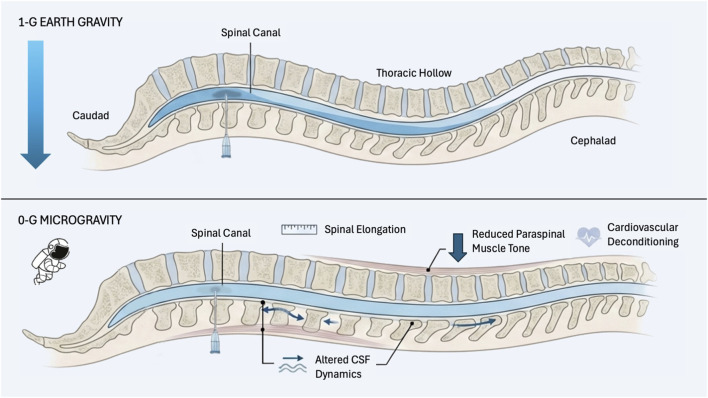
Conceptual model of spinal anaesthetic spread under terrestrial gravity and microgravity conditions. Under terrestrial gravity (top), intrathecal anaesthetic spread is shaped by baricity interacting with normal spinal curvature, producing predictable gravity-dependent distribution, with hyperbaric solutions settling in dependent regions. In microgravity (bottom), loss of a sustained gravity vector reduces density-dependent layering, shifting dispersion toward diffusion- and mixing-dominant mechanisms, while spaceflight-related changes in spinal geometry and physiology may further affect block characteristics and hemodynamic tolerance.

The curvature of the vertebral column is an important consideration as it affects the gravitational distribution of local anesthetic solutions. When a patient is supine on their back, the peak of the spine is L3 and the trough is at T5/6 (thoracic hollow). As seen in Figure 1, when injecting anesthetic at the highest point of the lumbar spine, the hyperbaric solution gravitates downwards into the thoracic hollow compared to a hypobaric solution which remains localised nearer the injection site. This finding is consistent with studies which have discovered that increased spread correlates with increased baricity. (Brown et al., 1980; Møller et al., 1984; Biboulet et al., 1993).

In contrast, in pregnant individuals the lumbar vertebrae are displaced posteriorly, causing a loss of the thoracic hollow and resulting in a reduced difference in the spread of anesthetic related to positioning or baricity (Russell and Holmqvist, 1987; Hirabayashi et al., 1995; Inglis et al., 1995; Richardson et al., 1998; Yun et al., 1998; Hallworth et al., 2005). While the mechanisms differ, this loss of spinal curvature and differences in baricity provide a useful analog to potential changes in microgravity. Caution must be given to avoid direct comparisons to this imperfect analogy, however, as pregnancy also involves increased intra-abdominal pressure, epidural venous engorgement, and hormonal modulation of neural sensitivity, factors not present in microgravity.

Neuromuscular diseases and vertebral column abnormalities, such as scoliosis, present a unique challenge for spinal anesthesia needle positioning and medication spread, which can be overcome through a variety of strategies (Gupta et al., 2012). Furthermore, abnormal spinal curvature may result in spinal anesthetic failure whereby a spinal anesthetic block was attempted but was not achieved at all or was achieved to an inappropriate extent for the planned procedure (Fettes, Jansson, and Wildsmith, 2009).

#### 1.2.2 The effect of temperature

Clinically, isobaric solutions often behave similarly to hyperbaric solutions, largely due to temperature-dependent reductions in viscosity after intrathecal injection. Experimental studies of hyperbaric bupivacaine demonstrate that warming reduces viscosity without significantly altering solution density (Arai et al., 2006). Correspondingly, both spinal canal models and clinical studies report higher sensory and motor block levels with bupivacaine warmed to 37 °C. (Stienstra et al., 1990; Callesen et al., 1991). While terrestrially relevant, temperature-viscosity behaviour in microgravity remains unknown and may not have an impact in this context.

## 2 Spaceflight physiology relevant to neuraxial anesthesia

In large part, the medical understanding of the effect of the space environment on the human body stems from research conducted in well-conditioned individuals. The health and fitness of astronauts is rigorously screened and closely monitored. The possible expansion of space tourism into ECMs may result in a new demographic of amateur astronauts of non-traditional backgrounds with pre-existing medical conditions. When determining the effect of the space environment on the multisystem physiologic changes in the human body, it is important to also consider that changes in response to acute adaptation to microgravity differ from chronic adaptations and that these changes may have variable relevance on neuraxial anesthesia depending on the duration or phase of the mission.

Furthermore, the evidence base informing our understanding of these changes varies substantially in physiological fidelity: parabolic flight experiments provide brief exposure to true weightlessness but cannot capture chronic adaptation; head-down tilt and bed rest analogues reproduce cephalad fluid shift without sustained unloading; and observational International Space Station data reflect long-duration exposure without procedural intervention, necessitating cautious extrapolation across complementary but incomplete models.

### 2.1 Musculoskeletal system

Delivery of spinal anesthesia to patients with altered curvature of the spinal column is challenging as it affects the spread of medication and degree of block achieved. In the context of prolonged exposure to the space environment, astronauts experience a loss of spinal curvature due to paraspinal muscle atrophy and spinal stiffening (Chang et al., 2016; Lazzari et al., 2021). Data collected by NASA reveals that, when exposed to microgravity, crewmembers experience a spinal elongation of up to 6% in seated height and up to 3% in stature (Young and Rajulu, 2020). Magnetic resonance imaging (MRI) scans of astronauts of the International Space Station demonstrate an average paraspinal muscle atrophy of 19% with no consistent change in intervertebral disc height (Chang et al., 2016). A notable reduction in supine lumbar lordosis was also observed, which decreased by an average of 11% (P = 0.019). This lumbar spine flattening was strongly associated with multifidus atrophy rather than intervertebral disc swelling, suggesting that loss of muscle mass significantly contributes to changes in spinal stature (Bailey et al., 2022).

During prolonged exposure to zero gravity, bone density loss and muscle atrophy are major concerns. The average loss of bone mineral density per month is 0.9% at the spine and approximately 1.5% at the hip (Baker et al., 2019). Regarding reversibility, countermeasures such as resistive exercise programs have been implemented to mitigate these effects. However, some spinal adaptations may persist after returning to Earth. Magnetic resonance imaging (MRI) assessments of six NASA crewmembers before and after a 6-month mission aboard the ISS indicate a significant reduction in the functional cross-sectional area of lumbar paraspinal lean muscle mass, dropping from 86% to 72%. While technical performance of spinal anesthesia in altered anatomy is feasible, the predictability of intrathecal spread in microgravity remains uncharacterized, particularly as musculoskeletal adaptations evolve over weeks to months and may only partially respond to countermeasures.

### 2.2 Cardiovascular system

Some of the most prevalent effects of microgravity involve the cardiovascular system, as fluid redistributes headward and is accompanied by an initial increase in cardiac output, and consequently stroke volume, as well as plasma volume. After a few days of adaptation, stroke volume and plasma volume decrease (Shibata et al., 2023). During prolonged spaceflight, autonomic dysfunction decreases systemic vascular resistance and inhibits the baroreflex, producing a system intolerant to orthostatic changes and blood loss (Norsk et al., 1979; Cooke et al., 2000; Agnew et al., 2004; Komorowski et al., 2016).

Therefore, the risk of cardiovascular collapse is a significant concern for procedures requiring spinal anesthesia (Komorowski et al., 2016). Terrestrially, hypotension following spinal anesthesia is driven largely by gravity-dependent venous pooling in sympathetically denervated vessels below the level of the block, resulting in reduced venous return and cardiac preload. In conceptual terms, this is analogous to producing a state of induced shock similar to relative hypovolemia, as might be seen in anaphylaxis. In microgravity, the abolition of hydrostatic pressure gradients eliminates this pooling mechanism, with venous return and cardiac preload maintained due to the cephalad fluid shift experienced in microgravity, with less blood being contained in the denervated vessels to begin with. In this case, resultant hypotension in microgravity is more likely to arise from overall reduced circulating blood volume, impaired autonomic reflexes, and limited cardiovascular reserve rather than redistribution of blood to dependent vascular beds.

Symptoms of hypotension can range from moderate in intensity, such as nausea and vomiting, to severe such as loss of consciousness (Sharma et al., 2024). In 2017, a double-blind randomised clinical trial investigated the effects of isobaric and hyperbaric bupivacaine 0.5% with fentanyl on maternal hemodynamics following spinal anesthesia for caesarean section. The incidence and duration of hypotension was lower in the group receiving isobaric bupivacaine when compared to the hyperbaric group receiving the same dose. As a result, the isobaric group required fewer vasopressors for the early correction of hypotension (Atashkhoei et al., 2017). Such research is strengthening our understanding of the influence of spinal anesthetic baricity on haemodynamic stability. However, the added element of microgravity may further complicate these findings. Thus, more research is needed to evaluate the potential for hemodynamic collapse in response to spinal anesthetic use in space.

### 2.3 Respiratory system

Spinal anesthesia can impair intercostal muscle function by blocking thoracic sympathetic and motor fibers, which on Earth may slightly reduce chest wall mechanics. In space, while the respiratory rate increases by almost 10%, the tidal volume decreases by about 15%, in a compensatory fashion to maintain minute ventilation (Prisk, 2005). Exposure to microgravity also leads to homogeneity of the ventilation-perfusion ratio, reducing the risk of atelectasis during ventilation (Prisk, 2005). However, there is otherwise little to no change in total lung volume and capacity measurements, nor physical dimensions after short or long-duration spaceflight (Ghani et al., 2023). These adaptations likely buffer or neutralize the respiratory effects of spinal anesthesia, provided the block does not ascend high enough to impair diaphragmatic function. Altered intrathoracic pressure dynamics during ventilation may secondarily influence CSF pressure transmission and contribute to the propagation of intrathecal medication through CSF flow dynamics, although the clinical relevance remains speculative both on Earth and in microgravity.

### 2.4 Neurological system

Exposure to microgravity leads to several neurological adaptations that could influence spinal anesthesia. One of the most notable is cephalad fluid shift, which may alter intracranial pressure (ICP) and cerebrospinal fluid (CSF) distribution, thereby affecting intrathecal drug spread and making baricity-based predictions less reliable than on Earth. Astronauts with optic disc oedema after long-duration missions have demonstrated elevated lumbar puncture opening pressures after return, suggesting that in some individuals ICP can remain raised post-flight (Alexander, 2012). In contrast, ground-based analogue studies using −6° head-down tilt (HDT) for 24 h show only a small, transient rise in ICP with subsequent normalisation, indicating that cephalad fluid shift alone does not necessarily produce pathological ICP elevations (Lawley et al., 2017). Brief periods of true weightlessness during parabolic flight have also been associated with decreases in both ICP and central venous pressure, likely due to changes in intrathoracic pressure, underscoring the importance of exposure duration and environment. (Videbaek and Norsk, 1997; Lawley et al., 2017). Finally, more recent model-based analyses in astronauts suggest that, on average, estimated ICP may even be lower after long-duration spaceflight in those without optic disc oedema, highlighting considerable inter-individual variability and uncertainty in true ICP behaviour in microgravity (Iwasaki et al., 2021).

Changes in CSF volume and flow add further complexity. Prolonged exposure to microgravity has been associated with modest increases in combined brain and CSF volume and enlargement of the lateral ventricles, although findings across studies are not entirely consistent (Lawley et al., 2017; Kramer et al., 2020). To achieve an equivalent terrestrial block in microgravity, increased CSF volume may require increased spinal anesthetic drug dosing, increased injected volume (with a concomitant decrease in drug concentration), or changes in the speed of injection. HDT experiments in healthy volunteers demonstrate reduced pulsatile CSF flow at the cervical level and increased jugular venous cross-sectional area, suggesting altered venous and CSF dynamics during simulated cephalad fluid shift (Marshall-Goebel et al., 2016; Zahid et al., 2021). While the contribution of CSF flow to anesthetic spread in terrestrial settings is currently unknown, one might hypothesize a reduction in block height from reduced mixing, or conversely an increase in block height from reduced CSF flow in the caudal direction.

Clinically, markedly elevated ICP remains an absolute contraindication to spinal anesthesia because of risks of herniation and CSF loss, and it may increase the likelihood of post-dural puncture headache and subdural haematoma (Acharya et al., 2001; Metterlein et al., 2010; Leffert and Schwamm, 2013; Didier-Laurent et al., 2021; Pinto et al., 2024). However, current evidence suggests that sustained pathological ICP elevation is uncommon in most astronauts, and elevated ICP alone is unlikely to be a uniform contraindication to spinal anesthesia in microgravity. Elevated ICP determination through symptomatic assessment or ultrasound evaluation of optic nerve sheath diameter may help guide decision-making in the absence of imaging capabilities such as CT or MRI, or direct, invasive ICP monitoring. Further work is needed to define how these CSF and ICP changes translate into block height, duration and complication rates in space.

## 3 Knowledge gaps and future directions

In this review, we recognize that neuraxial anesthesia may be operationally advantageous for spaceflight, though there remains little direct evidence describing how intrathecal drugs behave in microgravity. In general, there is a scarcity of publications examining all anesthetic modalities in microgravity conditions, but especially neuraxial anesthesia. Much of the existing literature relies on extrapolations from studies conducted under Earth’s gravity. Without significant advancements, the space medicine community will remain constrained by logistical challenges related to weight, volume, power, and crew training. Hence, it is critical to ensure a solid foundational understanding of anesthetic characteristics in microgravity so that space crew are best prepared to handle medical crises as they undoubtedly arise. Addressing this gap will require a tiered research strategy spanning complementary experimental platforms.

Near-term, low-cost studies should prioritize bench-top and computational approaches, including spinal canal phantoms and computational fluid dynamics models to examine CSF–drug mixing, injection-velocity effects, and pulsatile flow in the absence of gravity. These could be paired with parabolic flight experiments using intrathecal dye injection in physical spinal models to directly observe distribution under brief weightlessness.

Mid-term translational studies could employ animal models during parabolic flight or suborbital missions to assess intrathecal spread, motor block, and hemodynamic responses in true microgravity. Alternatively, intrathecal contrast dye myelography with radiographic imaging on parabolic flights could characterize distribution of a medication-analogue in human subjects.

Long-term investigations on orbital platforms such as the International Space Station would allow the evaluation of the complete temporal spectrum of intrathecal spread in spinal canal phantoms. Procedural feasibility could also be examined in this environment with pre-flight validation in microgravity analogues such as a neutral buoyancy lab. Finally, the safety and stability of medications used for spinal anesthesia would need to be established for long-duration missions that leave the Earth’s ionosphere.

Together, these complementary approaches would allow progressive refinement of mechanistic models and safety margins before any operational deployment of spinal anesthesia in exploration-class missions. In reality, there may be spaceflight scenarios in which the clinical imperative to intervene outweighs the uncertainty surrounding neuraxial anesthesia. As in other austere environments, technique selection in space will likely rely on graded risk–benefit assessment informed by available physiology, crew expertise, and procedural urgency rather than complete evidence. Thus, the goal of this research would not be to eliminate uncertainty but to characterize it, identify safety margins, and develop mitigation strategies that allow rational clinical decision-making when delay or non-intervention would pose greater risk. This becomes especially important if we are to consider protocol development for an increasing population of amateur astronauts with variable baseline health conditions, who may not appreciate the consequences of receiving an incompletely studied medical intervention and from whom it may not be possible to obtain true informed consent.

As humanity ventures into uncharted territories of space, the ability to provide timely and effective anesthesia will be necessary to ensure the safety and survival of crew members. Ultimately, evaluating the effectiveness of spinal anesthesia in space is not merely an academic pursuit; it is about ensuring the mission’s success during long-duration exploration and the crew’s safe return to Earth.
